# The novel concept of creating awareness about tuberculosis at the metro stations

**DOI:** 10.11604/pamj.2016.23.228.7127

**Published:** 2016-04-25

**Authors:** Sankalp Yadav, Gautam Rawal

**Affiliations:** 1 General Duty Medical Officer-II, Department of Medicine & TB, Chest Clinic Moti Nagar, North Delhi Municipal Corporation, New Delhi, India; 2 Attending Consultant-Respiratory Intensive Care, Max Super Specialty Hospital, Saket, New Delhi, India

**Keywords:** Chest clinic Moti Nagar, Delhi metro station, tuberculosis

## Abstract

Tuberculosis is an infectious disease and is a major health problem in developing countries like India. The disease is prevalent mainly in the underprivileged sections of the society. However, the same is not always true and even the well to do sections are also affected by this disease. The lack of knowledge in the masses and the communities is a factor that contributes largely to the spread of the disease. In such a scenario, there is always a need for new and innovative ideas to create mass awareness about tuberculosis.

## Commentary

Tuberculosis (TB) is a contagious disease which is now identified as a major public health concern in India, with millions of fatalities annually [1]. TB is caused by the acid fast bacillus Mycobacterium tuberculosis and is an important cause of mortality and morbidity worldwide due to its potential to affect any part of the human body including the lungs [2, 3]. India has the largest burden of TB in the world, having approximately one-fifth of the global incidence [4]. TB represents 3.75 percent (%) of India's ailment load and accounts for about four lakh deaths every year, making it one of the leading causes of death [1, 5]. The National Health Mission of India started the Revised National Tuberculosis Control Program (RNTCP) for the control of TB. The RNTCP by its virtue of regular communications to the clinicians and the general public has not only created a lot of awareness about TB in relation to its cause, symptoms, presentations and treatment, but also helped to understand and remove the various misconceptions and stigmas attached to it. Current circumstances require TB to be managed as a social issue and not as a therapeutic or even general wellbeing issue alone. The disease can be controlled by taking novel interventions aimed at various sections of the society [1]. WHO proclaimed TB a worldwide crisis in 1993, and the ‘Stop TB Partnership’ proposed a worldwide arrangement to stop TB, which plans to spare 14 million lives somewhere around 2006 and 2015 [1]. The stop TB strategy has a very important component, which is empowering people with TB, and communities [1]. In an immense nation like India, it is crucial to include each section of the society for viable counteractive action and TB control [1, 6]. The spots with high traveler loads like metro stations, bus stations, airplane terminals and the railroad stations are the fundamental focuses on that ought to be considered for this activity, subsequent to most of the populace is still not very much aware of about TB. This will help in widespread dissemination of the current knowledge and concepts about TB to the various social and economic sections of the society. The commuters are well disposed and perceptive to special health education messages and are more inclined to subsume the information and transfer it to other household members and friends. In this paper authors discuss about the novel concept of public awareness campaign against TB at one of the busiest metro station in New Delhi. The aim of this campaign was to increase the awareness and involvement of the masses towards the TB control program. Chest Clinic Moti Nagar (CCMN), a District Tuberculosis Centre (DTC), is the main point for all TB control activities in the district [4]. The District TB Officer (DTO) who is a Chief Medical Officer at the DTC has the overall responsibility of implementing DOTS and DOTS-Plus strategies of the RNTCP at the district level as per the program guidelines [4]. The Delhi DOTS Program has 25 Chest Clinics spread over whole of Delhi State. The CCMN is located in the West Delhi and it is one of the largest TB centers which cover a population of about 1.3 million. It has 24 DOT centers and 13 Designated Microscopy Centers (DMC's), under its control. The CCMN is working efficiently to cater to the needs of the ever growing number of TB patients. However, even after the regular awareness activities like the community meetings and other regular health education activities the number of new cases is still raising. Although, the recurrence rate is declining significantly, but even then the CCMN has had a heavy burden of the TB patients, including the drug resistant TB cases [4].

On the occasion of the World TB Day, 24^th^ March 2015 a number of activities were conducted all over the country [4]. The primary aim of these activities was to increase the awareness about the deadly condition, which is curable. The various activities included community meetings, rallies, puppet shows, educational material distribution, etc. Out of all these activities a novel concept was started under the supervision of the CCMN [4]. For the very first time in India, the CCMN started a public awareness initiative about TB for five days at the Delhi Metro Station, Moti Nagar. In the past, similar awareness campaigns were reported from all over the world. Even in India, a similar awareness campaign about the deadly swine flu was conducted [7]. In the year 2013, a twelve day campaign against the soaring rates of thalassemia was done in the UAE [8]. However, this is for the first time that such type of novel awareness activity related to TB was done in New Delhi at the metro station wherein, a medical officer and a DOT provider were actively involved in creating awareness for the general public. The Delhi Metro Station, Moti Nagar was chosen because it is one of the busiest metro station and has a huge number of commuters. This way the commuters got a chance to know about the disease. The commuters were informed about the symptoms, free diagnosis and treatment along with other necessary information about TB. They were given data pamphlets in two dialects, i.e. Hindi and English free of cost, which were plain as day [4]. The commuters were also encouraged to visit the CCMN, if in case they were having any symptoms of the TB, where they were clinically examined by the expert physicians for the disease. This whole awareness activity was conducted for five days, from 23.03.2015 to 27.03.2015 [4]. During these five days the response of the commuters was observed and it was found that the there was a gradual increase in the number of commuters stopping at the awareness station. Also, the patient inflow at the CCMN increased steadily with the prospective TB patients. The day and thus was fiscally very cheap [4]. The staff was really cooperative and was actively involved with the campaign. This campaign was also noticed by the WHO and the State TB Programme representatives and received recognition for such a novel concept [4].

The concept of starting an awareness campaign at a metro station is a very novel concept and should be started on a large scale. The mere conducting rallies on the roads on the World TB Day, distributing education materials about the disease or puppet shows to create awareness alone would not help, as the lack of information about TB is one of the important reasons for the spread of the disease [4]. The masses need to be informed about this disease at the busy centers like metro stations, railway stations, bus stands and alike, which is a low-cost affair and do not require much of the labor. The benefits are a lot and are not only confined to pro-actively finding the new cases, but will also make the people aware about the management and prevention strategies. The information about the common symptoms of TB and the availability of free diagnostic and treatment facilities will help in fostering the treatment seeking behavior [4]. Besides, the situation in a developing country like India, where a major part of the society earns less than one US dollar per day and wherein the government's annual health budget is lean there is a prime importance of such novel awareness campaigns, especially for the dissemination of the healthcare information to the masses and to achieve goals like health for all [9–12]. If all the TB centers throughout the country start such activities, then it will be possible to achieve the theme of the World TB Day 2015 which is, "Reach, treat, cure everyone". The state and the national governments and TB departments should encourage such activities for the betterment of the society. This will also help in controlling the ever-growing cases of MDR-TB, XDR-TB and TB-HIV patients. Besides, this concept can also be applied to create awareness about other diseases like dengue, swine-flu, malaria, leprosy, HIV-AIDS, etc.
